# Learning ultrasound assisted extraction through the use of sugar-coated chocolates

**DOI:** 10.1016/j.ultsonch.2025.107250

**Published:** 2025-02-08

**Authors:** Martina Novick, Nicolás Pérez, Sofía Barrios, Mariana Gonzalez, Patricia Lema

**Affiliations:** Universidad de la República Facultad de Ingenieria, Av. J. Herrera y Reissig 565 - Montevideo, Uruguay

**Keywords:** Education, Power ultrasound, Green chemistry, Extraction, Cavitation

## Abstract

Extraction of bioactive compounds from vegetal matrices is a dynamic field in pharmaceutical, cosmetic and food industry. Many of these compounds have high medicinal value. Ultrasonic assisted extraction (UAE) becomes an interesting alternative to reduce both the amount of chemical solvents and the processing time.

The aim of the present paper is to introduce, intuitively, the physical mechanisms involved in ultrasonic extraction for non-specialists.

Two different mechanisms, cavitation and acoustic streaming, can be easily experimentally shown. The proposed experiment is the extraction of the sugar cover in sugar-coated chocolates. Extraction is analyzed using video tools to obtain a quantitative evolution of the process. Graphical results and videos are a useful tool for showing the basis of ultrasonic extraction in several levels from primary school to university students. In the event that the necessary ultrasound equipment is not available, we hope that the videos available in the supplementary material will be useful for teaching the topic.

## 1 Introduction

**Fig. 1:**
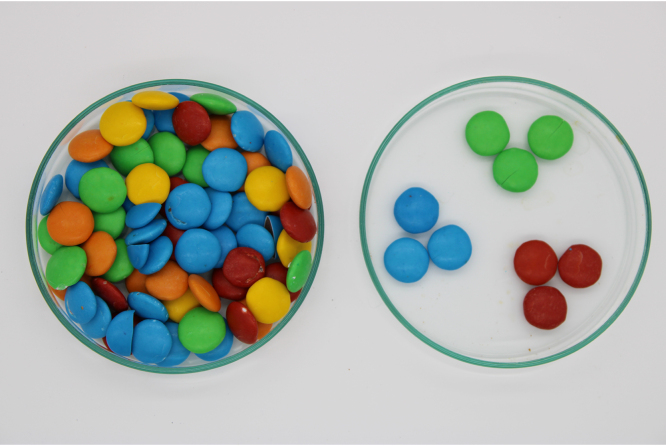
Reference image of sugar-coated chocolates. See the variety of colors and the aspect of the surface.

Dissemination and teaching of science and technology are essential tasks for researchers and university professors. We often focus on providing thorough and formal explanations for a specialized audience. However, making scientific knowledge understandable to everyone is equally important. This work stems from one of these efforts. Our research group focuses on industrial applications of ultrasound, particularly ultrasound applied to the food industry [1], [2], [3]. Annually, the Faculty of Engineering organizes a public outreach event [4], and one of the main challenges is to demonstrate the fundamentals of our work to an audience ranging from school-aged children to students interested in pursuing postgraduate studies at the university. Within the industrial applications of ultrasound, we have selected the use of ultrasound in extraction of natural products for presentation [5], [6], [7]. Meanwhile, our department teaches a postgraduate course, “Experimental Ultrasound Techniques” that is part of the training of master and doctoral students in Electrical Engineering, Civil Engineering and Chemical Engineering [8]. At this level, experiments must be complemented with quantitative procedures that allow evaluating the effects of ultrasound on the product to be treated. The aim of the present work is to present a simple but very illustrative experiment used by our group to show the main mechanisms of power ultrasound in extraction of natural products. The experiment consists in the extraction of the sugar coating in candy chocolates. Fig. 1 shows the variety of colors that these chocolates present. The use of a popular candy like the sugar-coated chocolates can be attractive for children and the general public. The main speech to introduce the experiment could be something like this: *Here we have an example of how the extraction of natural products works. The extraction consists in obtaining some compound from the vegetables using a solvent and temperature in most cases. An everyday example is when we make tea, where polyphenols and minerals are extracted using hot water. Power ultrasound can help in such kind of process. As an example you can see how the ultrasound accelerates the extraction in the case of the sugar cover in these chocolates.* In this experiment we can show how the ultrasound changes the temporal scale of the experiment form a few minutes in the case of water extraction to a few seconds in the case of ultrasonic assisted extraction. An additional advantage is that temperature sensitive compounds can be extracted, since ultrasonic extraction can be performed using low temperature levels. In the next section, we include frequently asked questions by the public who attend these experiments. The idea is to give simple and intuitive answers about the main aspects of ultrasound-assisted extraction. For university undergraduate and graduate levels, experiments can be evaluated quantitatively using image analysis. In the Materials and Methods section, we include the basic experimental setup and the tools used for processing the video images. As attached material, two original videos and an example of data processing are included to visualize the results. In the results section we include the color evolution curves as a function of time for conventional and ultrasound-assisted extraction. This allows us to compare the different speeds of the process. Three sets of videos taken with samples of different colors are included as attached material.

## 2 Frequent questions

In this section, we address common questions often posed by the public following the presentation of this experiment. We provide straightforward answers suitable for a general audience, while interested readers are encouraged to consult the references for more detailed information.

### 2.1 What is ultrasound?

Ultrasound is a mechanical wave that propagates through a medium. The word ultrasound is used to highlight that it is above the frequency audible to the human ear. This limit is accepted as 20 kHz. In the case of the extraction process, these are pressure waves that propagate in a liquid medium and interact with vegetable elements in suspension. Ultrasound applications can be divided into two large groups: inspection ultrasound and power ultrasound. In the case of inspection, for example for medical imaging ultrasound, the energy levels are low and do not produce alteration effects in the media. The extraction process requires power ultrasound; in this case, the wave produces appreciable changes in the medium [9], [10].

### 2.2 How is ultrasound generated?

**Fig. 2:**
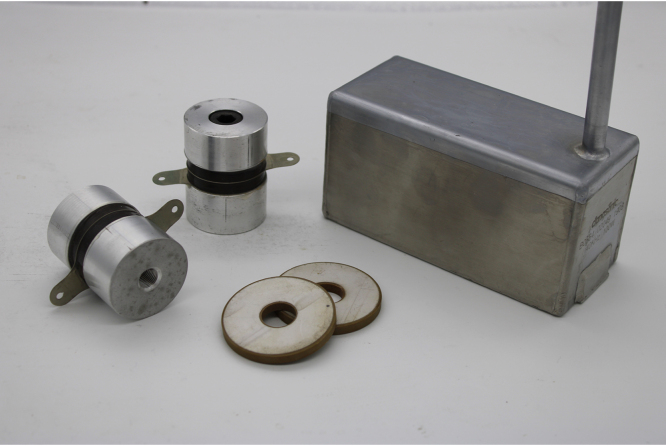
Langevin transducers and piezoelectric ceramics used to generate ultrasound.

Just as sound is produced by speakers, in the case of ultrasound we have elements called ultrasonic transducers that produce high-frequency, high-power vibration. These transducers are generally based on the piezoelectric effect, where a voltage produces a mechanical deformation. This effect is reversible, a mechanical deformation produces a voltage, functioning as a microphone. In the case of power transducers, a Langevin type structure is used, where two metal pieces form a tuned resonator together with the piezoelectric material [11]. Fig. 2 shows piezoelectric ceramics and an example of a Langevin transducer used in commercial devices. Here we can see the device used to generate the ultrasound. Inside it we have two Langevin transducers that can deliver up to 50 W each. The central part of the transducers is formed by two ring-shaped piezoelectric ceramics as seen in the figure.

### 2.3 How does ultrasound work in the extraction process?

**Fig. 3:**
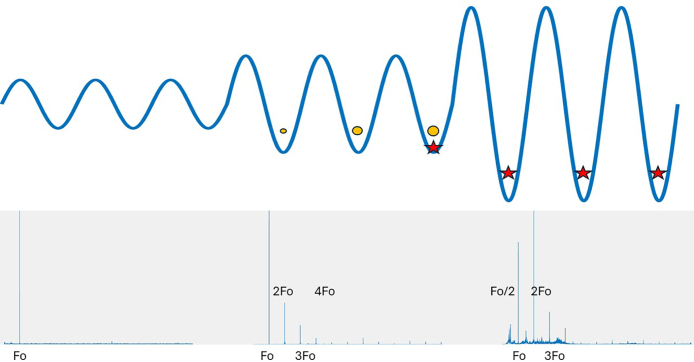
Schematic representation of the cavitation process. When acoustic intensity is low, no cavitation occurs (left images). When acoustic intensity is medium, stable cavitation occurs (center images). When acoustic intensity is high, transient cavitation occurs (right images).

There are three main mechanisms by which ultrasound helps in the extraction process. These mechanisms are cavitation, acoustic transmission, and heating. It is generally accepted that the main contribution of power ultrasound to extraction is due to cavitation. Cavitation consists of the local generation of pressure peaks produced by the implosion of bubbles within the liquid. The sound wave produces a pressure that is lower than that of vapor in the negative half cycle, allowing the growth of a gas cavity. This cavity implodes, producing a pressure peak that has destructive effects when it occurs on a surface. A common experiment to show the destructive effects of cavitation is the use of a sheet of aluminum foil. In the case of extraction, cavitation achieves the rupture of the vegetable tissues. Fig. 3 shows a schematic representation of the effect of cavitation. For low pressure amplitudes (image on the left), bubbles are not generated in the liquid. When the pressure amplitude increases, a stable cavitation regime occurs. Here, the bubbles oscillate until they collapse (central image). Finally, when the acoustic field is intense, transient cavitation occurs, where the bubbles collapse violently (image on the right). This effect can be seen (heard) in the acoustic field generated in the liquid by the bubbles. In the lower part of the figure we see the emission spectrum of the bubbles received by a hydrophone. A hydrophone is a special type of transducer that allows the acquisition of pressure signals within a liquid. Here, Fo indicates the transducer emission frequency. Note that depending on the intensity, harmonics, subharmonics, and white noise are received in the acoustic signal.

Pressure gradients within the fluid produce macroscopic movement of the liquid or acoustical streaming. This movement is unstable because the surface of the liquid moves. It is a mechanism similar to mechanical agitation in the case of conventional extraction. Finally, all the energy transferred by the transducer to the liquid causes a temperature rise in the liquid. The effect of using a 100 W ultrasound transducer is the same as that of using a 100 W heater from a thermal point of view. Energy is transferred irreversibly as temperature of the liquid medium. [5]

### 2.4 What are the advantages and disadvantages?

As advantages we can mention that the three mechanisms contribute to the extraction process. This allows in many cases the extraction of a greater quantity of product, reducing the use of chemical solvents and process time. The reduction in the use of solvents allows us to call this technique as green chemistry. An additional advantage is the efficiency of heat transfer to the medium. In traditional processes, the edges are heated and diffusion occurs towards the interior of the medium, which generates higher temperature at the edges than in the interior. In the case of ultrasound, the energy is transported by a wave, which penetrates the liquid and heats the interior volume uniformly. Disadvantages include the difficulty in scaling the results, the excessive energy consumption in some cases and the greater complexity of the devices on an industrial scale. In each case, the pros and cons must be evaluated, balancing costs, times and energy performance. [12].

## 3 Materials and methods

### 3.1 Experimental setup

**Fig. 4:**
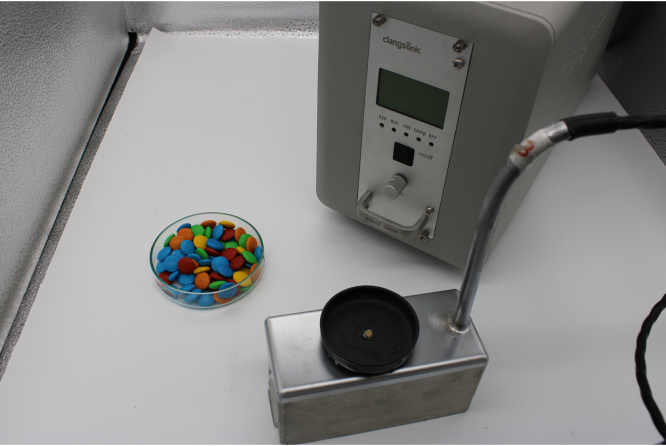
Experimental setup. A commercial photographic set up is used to avoid light reflections.

The experimental setup consists of a 100 W transducer designed for generic ultrasound applications (Clangsonic TU0340-F21, 48 kHz, 300 W). The device allows three simultaneous 100 W tests to perform triplicate evaluations on extraction samples. We use a glass sample holder to center the candy used in the test. To avoid movement when filming the video, both the sample holder and the candy are glued with cyanoacrylate. To avoid light reflections and improve the quality of the videos we use a portable photographic set. Also, the sample holder is painted black. The videos are taken with a digital camera (Canon EOS 2000D) using manual zoom and acquiring 25 frames per second. Fig. 4 shows the ultrasonic transducer and the photographic set up.

The experiments can be divided into three steps: first, the sample holder where the candy has been stuck is filled with water (this part is omitted in the attached material). Then 40 s are left to observe the speed of conventional water extraction at that temperature. Finally, the ultrasound is turned on and left to act for 40 s. Three videos containing the raw videos are placed in the attached supplementary material, one for each color of candy. To simplify image analysis, the videos are cropped to one minute in length, including only the section of the image that contains the ultrasound transducer. During the first 25 s, extraction occurs only with the action of water, while in the remaining time the action of ultrasound can be seen.

### 3.2 Video analysis

To obtain quantitative results, we use video image processing. This processing is not very sophisticated and can be performed by non-specialists. Any software with video analysis and numerical calculation capabilities can be used, for example Python, Octave or Matlab. We give a prototype of the processing functions in the attached material using Matlab R2018. The raw videos available are: candyred.mp4, candygreen.mp4 and candyblue.mp4.

**Fig. 5:**
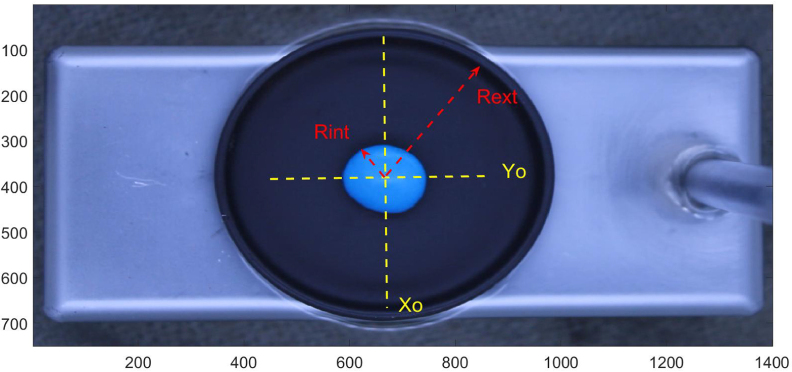
Position of center and radius for blue candy.

For each file we must give the position of the center of the candy and the radius of the circle where the analysis is performed. This radius is slightly smaller than the sample holder to avoid reflections at the edges. Obtaining these values can be done graphically using the ginput (graphical input from mouse) command or similar image processing tool. For simplicity, we have placed the values in the sample script. The reader can use the videos and processing programs without worrying about the determination of these parameters. The processing program applies a mask in order to analyze only the region of the image around the candy. The mask multiplies by one in the region of interest and by zero outside it. Computer images encode color information differently. A grayscale image associates a number (usually 8 bits 0–255) to each pixel in the image. For color there are different codes, RGB being one of the most used. Here, each point has three 8-bit numbers corresponding to the amount of red, green or blue that the color of each pixel has. As an example, black is (0,0,0) and white is (255,255,255). A computer image can be seen as a matrix of nxm pixels containing the RGB information of each position. For each frame of the video, the average value of the R, G and B components of the region selected for analysis is obtained. From a didactic point of view, this analysis allows to introduce an important color image analysis tool in a simple way. Fig. 5 shows the raw image, the center and the radius for the blue candy.

In the attached files we include the CandyProcessing.m, this script runs the complete data processing.

We include the videos with the mask used for color calculation. These videos are named as: maskred.mp4, maskgreen.mp4 and maskyblue.mp4.

### 3.3 Extraction speed calculation

From the data obtained from video analysis, extraction speed for both conventional extraction and ultrasonic assisted extraction can be calculated. Firstly, the mean value of the RGB components for the color of each type of candy must be determined at time cero of the experiments. This represents the color of the intact candy, and can be used to normalize the averaged RGB values at each experimental time. Therefore, averaged RGB values with time can be expressed as a percentage of these initial RGB values. After normalization, RGB percentages can be plotted against extraction time for each experiment. A linear regression can be performed both for the first 25 s of the experiment (corresponding to conventional water extraction) and for the remaining extraction time (corresponding to ultrasound assisted extraction). From the slope of the linear regression, extraction speed can be calculated as percentage per second.

## 4 Experimental results

Mean values of the RGB components for each candy color were calculated at time cero of experiments. For example, in the case of the blue candy we see it as blue, but the RGB components are not a pure blue, they are a combination of Red, Green and Blue.

The components presented in Table 1 give us the level of color that is expected to be extracted from each candy. Note that in addition to the color coverage there is a layer of sugar underneath. The sugar will appear in the images as close to white, producing tones in all three RGB components.

**Table 1:** RGB components of sugar coated candies.

Candy	[R G B]
Red	[145 35 22]
Green	[76 178 71]
Blue	[37 142 192]

**Fig. 6:**
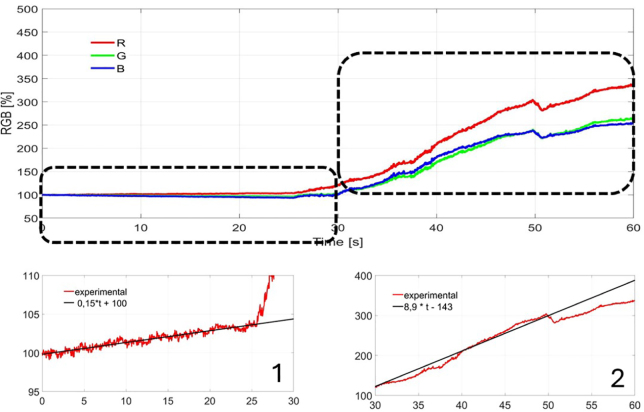
Extraction for the red candy. 1 Linear adjust for R component in first 25 s. 2 Linear adjust for R component in second 25 s window.

**Fig. 7:**
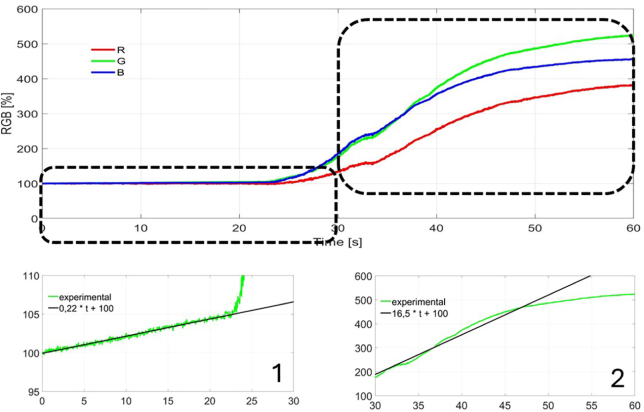
Extraction for the green candy. 1 Linear adjust for G component in first 25 s. 2 Linear adjust for G component in second 25 s window.

**Fig. 8:**
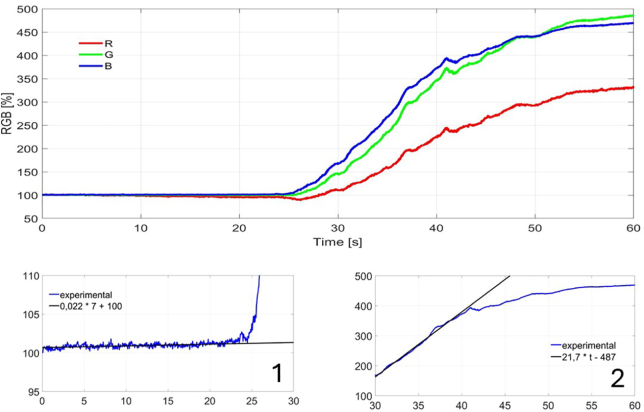
Extraction for the blue candy. 1 Linear adjust for B component in first 25 s. 2 Linear adjust for B component in second 25 s window.

For each candy, the speed of the process for both conventional extraction and ultrasound-assisted extraction was determined. The quantitative results presented in Fig. 6, Fig. 7, Fig. 8 are normalized by the color of intact candy and are expressed as a percentage of this value. The lower left subplot shows the evolution and a linear fit for the first 25 s of extraction in water. The lower right subplot shows the evolution of ultrasonic assisted extraction.

As can be observed in the figures, the speed of extraction in the conventional extraction is clearly lower than the speed obtained using ultrasound. The process is quite similar for each color (see Table 2).

We include the videos with the results of the extraction evolution for each color. These videos are named as: redevolution.mp4, greenevolution.mp4 and blueevolution.mp4. The results are expressed as a percentage of the first value for each video.

**Table 2:** Extraction speed for conventional and ultrasonic assisted extraction.

	Conventional [%/s]	Ultrasonic assisted [%/s]
Red	0,15	8,9
Green	0,22	16,5
Blue	0,022	21,7

## 5 Conclusions

In this work we present a simple experiment to show the mechanisms involved in ultrasonic assisted extraction. Influence of cavitation and ultrasonic streaming can be observed directly in the videos. Thermal effects of ultrasound can be easily incorporated in the experiment simply using a thermometer and observing the temperature evolution. However, we think that this effect requires longer times to be observed and adds complexity to the experiment. The experiment can be used at two different levels. Firstly, it serves as a straightforward method to show the effects of ultrasound to children and the general public. Secondly, for graduate and university students, we can conduct image analysis to quantify the extraction speed of both conventional and ultrasonically assisted extraction methods. In the attached supplementary material we include the original videos to carry out data processing or present in the classroom. A prototype of a processing program using numerical calculation software is also given. The prototype only requires having the videos and the program in a folder on the computer and having Matlab 2018 or higher installed. We also include the videos obtained in the processing so that they can be used in class without the need to perform numerical calculations. We hope that the material presented here can contribute to the dissemination and teaching of ultrasound techniques, in particular, the use of power ultrasound for the extraction of valuable compounds from vegetable matrices.

## CRediT authorship contribution statement

**Martina Novick:** Validation, Investigation, Data curation, Conceptualization. **Nicolás Pérez:** Writing – original draft, Supervision, Methodology, Conceptualization. **Sofía Barrios:** Writing – original draft, Methodology. **Mariana Gonzalez:** Methodology, Conceptualization. **Patricia Lema:** Supervision.

## Declaration of competing interest

The authors have declared no conflict of interest.

## Appendix A Supplementary data

Video S1 Collection of videos for educational purposes.
